# Understanding and leveraging uncertainties in autosegmentation for radiotherapy

**DOI:** 10.1093/bjrai/ubaf013

**Published:** 2025-07-08

**Authors:** Stine Sofia Korreman, Jintao Ren

**Affiliations:** Department of Clinical Medicine, Danish Center for Particle Therapy, Aarhus University, DK-8200 Aarhus, Denmark; Department of Clinical Medicine, Danish Center for Particle Therapy, Aarhus University, DK-8200 Aarhus, Denmark

**Keywords:** radiotherapy, autosegmentation, deep learning, uncertainties, interpretability

## Abstract

This article examines the role of uncertainties in autosegmentation for radiotherapy, which has shown promise in improving consistency, accuracy, and efficiency over traditional manual delineation. Autosegmentation, however, introduces challenges due to uncertainties that arise from factors such as limited training data and models. A nuanced understanding of these uncertainties is essential, as they directly impact clinical decisions and patient outcomes. We explore the sources of uncertainties and how they may be quantified, highlighting the impact on different levels of the segmentation process and discussing the implications of imperfect predictions. Practical applications are proposed, such as uncertainty maps to guide manual adjustments and flagging mechanisms to support quality assurance. We finally address the limitations of these approaches, including computational burdens and risks of information overload. Meaningful uncertainty quantification in autosegmentation holds significant potential to enhance clinical workflows, build trust in artificial intelligence-based tools, and ultimately improve patient care in radiotherapy.

In radiotherapy, autosegmentation has emerged as a powerful tool to address the limitations and challenges related to manual segmentation. Autosegmentation has been demonstrated to reduce time consumption in delineation and to have the potential to enhance consistency and accuracy, thereby contributing to safer and more effective patient care.^1^^,^^2^ However, autosegmentation algorithms exhibit uncertainties stemming from multiple factors including limitations in underlying data and models. Recognizing, quantifying, and utilizing these uncertainties is crucial, as segmentations directly influence clinical decision-making, and thereby patient outcomes.

## Sources of uncertainty

Autosegmentation uncertainties arise from a variety of factors, and understanding the origins and manifestations of these uncertainties is the first step towards developing better tools and processes for handling them.

When analysing and accounting for uncertainties in autosegmentation models, a useful distinction can be made between epistemic and aleatoric uncertainties.^3^ Epistemic uncertainty reflects a model’s lack of knowledge, arising from limitations in architecture or insufficient training data - like underrepresentation of rare pathologies. It can be mitigated through exposure to more representative and diverse data. Aleatoric uncertainty, on the other hand, stems from inherent data ambiguity, such as inter-observer variability, imaging noise, or artefacts. This type is stochastic and cannot be reduced through additional data or architectural changes. Table 1 outlines typical sources of both uncertainty types in autosegmentation. In practice, predictive uncertainty captures the combined influence of epistemic and aleatoric uncertainties, providing a comprehensive measure of a model’s confidence in its predictions.^3^

**Table 1.** Sources of aleatoric and epistemic uncertainty in autosegmentation in radiotherapy.

Category	Source of uncertainty	Type	Explanation
Imaging	Low signal-to-noise ratio (SNR)	Aleatoric	Random noise makes boundary identification difficult, especially in soft tissues.
	Motion artefacts (eg, due to breathing or swallowing)	Aleatoric	Introduces random spatial distortions that obscure anatomical boundaries.
	Low resolution or poor contrast	Aleatoric	Limits ability to resolve fine structures, leading to boundary ambiguity.
	Scanner variability or protocol differences	Epistemic	Model trained on a specific scanner or protocol may not generalize to others.
Annotations	Inter-observer variability	Aleatoric	Clinician interpretations vary, introducing noise into the ground truth labels.
	Incomplete or inconsistent labelling in training data	Epistemic	Leads to uncertainty in model learning and generalization.
	Rare pathologies not annotated or inconsistently labelled	Epistemic	Model has limited exposure and thus poor performance on underrepresented cases.
Anatomy & Pathology	Anatomical variation across patients	Aleatoric	Natural variability in human anatomy introduces inherent segmentation ambiguity.
	Rare or complex anatomical presentations	Epistemic	Model fails to generalize due to lack of similar examples in training.
	Tumours invading uncommon regions (eg, into bone/spine)	Epistemic	High model uncertainty due to unfamiliar pathological spread.
Workflow	Imaging modality misregistration (eg, CT-MRI alignment)	Aleatoric	Misalignment introduces voxel-level ambiguity in multi-modal segmentation.
	Temporal differences in imaging (eg, tumour progression)	Aleatoric	Images captured at different times may not align with prior segmentations.
	Population shift between institutions	Epistemic	Model may not transfer well across institutions due to demographic or equipment differences.

Epistemic uncertainty tends to manifest at the patient or subvolume level, particularly when the model encounters presentations that differ significantly from its training data. For instance, if a model is trained on a data set of head and neck cancers which consist of squamous cell carcinomas of the oropharynx, inference for rare histologies or adjacent tumour locations may trigger high uncertainty and segmentation errors.^4^ The same may be the case for an anatomically rare and complex cases, such as tumours invading the spine. Anatomically intricate structures, rare pathologies, or unique patient-specific anomalies are generally particularly vulnerable to false positives/negatives.

On the other hand, aleatoric uncertainty is more pronounced at the voxel level, where ambiguous image features lead to unclear boundaries. These limitations are often rooted in the imaging modality itself - low signal-to-noise ratio, poor contrast, or limited resolution - contributing to interobserver variability among annotators and lack of definitive ground truth.^5^^,^^6^ This complicates evaluation and introduces label noise, potentially biasing model training. Addressing such variability through consensus labels, uncertainty-aware learning, or multi-annotator datasets is essential for building robust, generalizable models.^7^^,^^8^

Utilizing multiple imaging modalities would often help reduce uncertainties by providing complementary information.^9^ However, each modality also has specific limitations that contribute to uncertainties and combining modalities may increase the combined uncertainty magnitude. Additionally, registration errors between modalities can introduce conflicting voxel-level information. Strategies like image registration, normalization, and modality fusion can help harmonize cross-modality inputs and reduce inconsistency, but residual variability often remains.^4^

While the distinction between aleatoric and epistemic uncertainty is well-established in research, it is often approximate in practice, as both types of uncertainties can coexist and are challenging to disentangle or validate separately.^10^^,^^11^ Recent studies also question the effectiveness of this decomposition, highlighting its theoretical and practical limitations.^12^ Moreover, direct clinical utility may be limited, and a unified predictive uncertainty may be more practical for clinicians.^11^

## Consequences of imperfect model predictions

A series of consequences arise when model outputs are unreliable, ultimately having the potential to affect patient outcomes, clinician workload, and the overall credibility of the technology. If a model is biased due to its training data or is inconsistent in its performance across cases, it may yield uneven quality in treatment planning. Bias or inconsistencies in segmentation quality can lead to suboptimal dose delivery, risking both treatment efficacy and patient safety.

When predictions lack reliability, clinicians may need to spend extra time correcting or validating model outputs. Unreliable model outputs may therefore translate to increased manual intervention from clinicians. Rather than streamlining workflows, poor autosegmentation models can become burdensome, adding to clinicians’ already substantial workloads, potentially leading to treatment delays, particularly in high-throughput clinical environments.

Finally, repeated instances of incorrect or biased predictions can undermine clinicians’ trust in autosegmentation tools. Lack of trust reduces the likelihood of technology adoption, leading to missed opportunities for early intervention and underutilization of tools that could otherwise enhance clinical care.

Uncertainty quantification can be used to provide tools to enhance interpretability and transparency in an effort to mitigate these potentially detrimental consequences of imperfect models.

## Quantifying meaningful uncertainties

Various predictive uncertainty quantification methods, including Monte Carlo dropout, Deep Ensemble, test time augmentation, and conformal prediction provide ways to estimate uncertainty in model outputs.^13^ A key question is whether these uncertainty quantifications are meaningful in a sense that they may aid clinicians in interpreting segmentation reliability.^14^

Investigation of various uncertainty quantification methods has shown differences between methods, but with an overall promising capability of estimating confidence levels in segmentation predictions, offering a path forward for making model outputs more interpretable.^13^ Reliability diagrams and calibration metrics can evaluate how well uncertainty estimates match actual model performance by comparing predicted confidence scores with real outcomes, helping determine if a model’s uncertainty is well-calibrated and offering a coarse but useful signal of reliability under potential distribution shifts.

**Figure 1.**
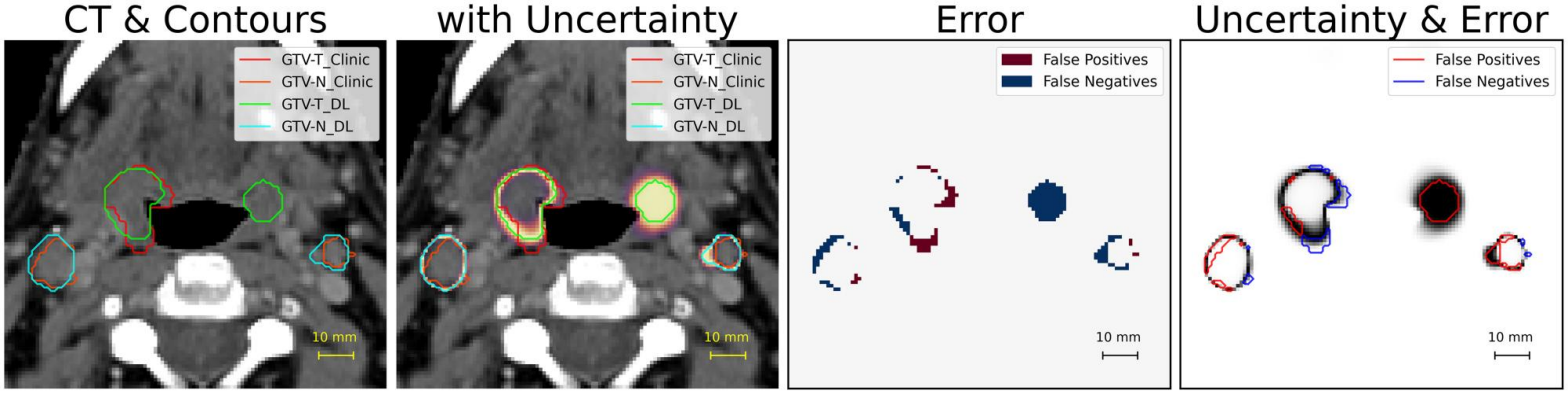
Segmentation and uncertainty visualization for deep learning autosegmentation of head and neck tumours. From left to right: CT with clinical ground truth (GTV-T: red, GTV-N: orange) and deep learning predictions (GTV-T: green, GTV-N: cyan); CT overlayed with uncertainty and contours; error map (false positives: dark red, false negatives: navy); and uncertainty map with error contours. Uncertainty quantification was performed using a combination of Deep Ensembles, Monte Carlo dropout, and test-time augmentation.

Meaningful uncertainty quantification can thus serve as an indicator of potential errors, revealing regions that may require closer examination.^13^^,^^15^^,^^16^ Confidence calibration - aligning prediction confidence with actual model accuracy - enhances interpretability by ensuring that confidence levels reflect true reliability. By examining uncertainty quantification along with segmentation prediction, clinicians can more accurately identify regions needing adjustment (Figure 1).

In addition, robust uncertainty quantification plays a crucial role in detecting out-of-distribution (OOD) instances arising from deviations in imaging protocols, scanners, anatomical regions, or clinical presentations.^10^ Given the variability of sources, OOD detection is context-dependent and better understood as a spectrum rather than a binary state. Uncertainty quantification methods may allow systems to flag potential OOD cases, enabling clinicians to anticipate when a model may fail. However, common uncertainty quantification methods, such as Monte Carlo dropout, can fail to reliably distinguish OOD scenarios, particularly under subtle domain shifts.^17^ In addition, calibration tends to degrade beyond training distribution, leading to overconfident predictions.^18^ Combining uncertainty estimates with dedicated OOD detection techniques may improve the identification of unfamiliar inputs.

## Practical applications of uncertainties in clinical workflows

Once uncertainties are quantified, the next challenge is the practical adoption of these models in clinical settings, to improve clinical workflows and decision-making.

First, uncertainty quantification can enhance the interpretability of segmentation outputs by indicating where model predictions may be less reliable. Uncertainty maps may serve as visual tools, highlighting specific subvolumes or regions that require closer inspection or manual adjustment, guiding clinicians in interactive and targeted refinement of high-stakes regions (Figure 1). Such visualizations help prioritize clinician attention and ensure that regions exhibiting predictions with high uncertainty receive appropriate scrutiny.^19^

Additionally, transparency in model uncertainty can foster clinician confidence and provide a foundation for uncertainty-aware decision-making. Uncertainties can potentially even guide active learning strategies, whereby the model learns from clinician feedback on uncertain regions. This active refinement process improves model accuracy and allows for personalized contouring suited to individual patients.^20^

In scenarios where models are adapted across different institutions or patient groups, traditional approaches focus on segmentation performance and applying transfer learning when it falls short. However, it is equally important to ensure the quality of uncertainty estimates. If these are unreliable, additional calibration may be required to improve uncertainty accuracy of an adapted model. This dual focus on segmentation and uncertainty quality enhances model reliability, supporting broader clinical adaptability and confidence across varied patient populations.

## Key limitations and future perspectives

While uncertainty quantification offers clear advantages, it also comes with limitations. Not all model errors are detectable, and excessive uncertainty visualizations can overwhelm clinicians through information overload. For instance, uncertainty within the range of inter-observer variability can in some cases be considered clinically acceptable but may still be shown in visualizations. Moreover, some methods - like Monte Carlo Dropout or ensembles - are computationally intensive, posing challenges for real-time or resource-limited settings.

As autosegmentation models evolve to incorporate multi-modal inputs - such as clinical data and text prompts, especially through language-vision architectures^21^ - interpretability tools like SHAP^22^ and LIME^23^ gain importance alongside visual methods like probability, uncertainty, and saliency maps (eg, GRAD-CAMs^24^). Future systems may combine uncertainty estimates with explainable artificial intelligence (AI) tools to deliver more actionable outputs, highlighting high-uncertainty regions or generating priority scores and summary reports to support efficient clinical decision-making.

However, as these technical advancements progress, they also give rise to broader discussions: While uncertainty quantification holds promise for more reliable AI, it introduces new challenges related to responsibility and patient safety. For instance, who is responsible if uncertainty is flagged but overlooked, or when a model fails to flag an error? Should uncertainty maps and clinician response be stored in the medical record? Recognizing these challenges, it becomes essential to balance innovation with accountability, and to design trials to test actual clinical benefit of advanced autosegmentation tools.

In summary, while autosegmentation has become an essential tool in radiotherapy, the uncertainties inevitably inherent in model predictions underscore the need for robust uncertainty quantification practices. With careful integration into the workflow, this offers a path towards more reliable, interpretable autosegmentation models that address the complexities of clinical applications and improves both patient outcomes and clinician workflows.
